# Discovery of Drug-like Inhibitors of the Human Caf1/CNOT7 poly(A)-Selective Nuclease Using Compound Screening

**DOI:** 10.3390/biom15111563

**Published:** 2025-11-06

**Authors:** Ishwinder Kaur, Lubna Hashmi, Peter M. Fischer, Gerlof Sebastiaan Winkler

**Affiliations:** School of Pharmacy, Biodiscovery Institute, University of Nottingham, University Park, Nottingham NG7 2RD, UK; ishwinder.kaur@ntu.ac.uk (I.K.);

**Keywords:** Caf1, CNOT7, CNOT8, mRNA, deadenylase, inhibitor, nuclease, gene regulation, compound screening

## Abstract

The human Ccr4–Not complex is a central regulator of post-transcriptional gene regulation, impacting on translation and mRNA degradation. In mRNA degradation, Ccr4–Not participates in the shortening of the mRNA poly(A)-tail via two catalytic subunits. The Caf1 nuclease is encoded by the highly similar paralogues CNOT7 or CNOT8. In addition to its poly(A)-specific ribonuclease activity, this subunit also provides a structural role by binding Ccr4, the second catalytic nuclease subunit encoded by the paralogues CNOT6 or CNOT6L. To facilitate investigations into the roles of the Caf1 subunit, and to complement genetic tools, we set out to identify inhibitors of the enzymatic activity of Caf1/CNOT7. To this end, we screened a library of 10,880 chemically diverse, drug-like compounds using a fluorescence-based biochemical assay. This effort led to the discovery of 15 inhibitors of Caf1/CNOT7 with biochemical IC_50_ values below 25 μM. Molecular docking was performed to explore potential binding modes of these compounds. The compounds reported here may be useful to differentiate between catalytic and non-catalytic roles of Caf1/CNOT7. In addition, they may be valuable starting points for the development of more potent inhibitors of the Caf1/CNOT7 poly(A)-selective ribonuclease.

## 1. Introduction

The eukaryotic Ccr4–Not complex plays an important role in post-transcriptional gene regulation by influencing translation and mRNA degradation [1,2,3,4,5]. In yeast and human cells, the complex is the main enzyme involved in shortening of the poly(A) tail (deadenylation) and can directly interact with the translating ribosome [6,7,8].

In mRNA degradation, the Ccr4–Not complex collaborates with Pan2-Pan3 in mRNA deadenylation, which is the first and often rate-limiting step in cytoplasmic mRNA degradation [3,9,10,11]. The complex contains two catalytic subunits with poly(A)-selective ribonuclease activity. The Caf1 subunit belongs to the RNAse D superfamily of nucleases and provides the complex with 3′-5′ exoribonucleolytic activity selective for poly(A) sequences [12,13]. In addition, the Caf1 subunit has a structural role that facilitates binding of the second poly(A)-selective nuclease, Ccr4. The Ccr4 subunit binds Caf1 via a leucine-rich repeat (LRR) domain [14,15,16], while its catalytic activity is provided by an endonuclease–exonuclease–phosphatase (EEP) domain [17]. Currently, there is conflicting evidence as to whether the Caf1 and Ccr4 subunits collaborate during deadenylation, or whether they have specialised functions. Some studies reported possible inter-dependence of the two catalytic subunits [18,19,20], while other reports suggested that the catalytic subunits act independently [21,22].

In metazoan cells, the Caf1 and Ccr4 subunits are each encoded by two paralogues, CNOT7/CNOT8 and CNOT6/CNOT6L, respectively [23]. In human and mouse cells, the paralogues have overlapping roles and their functions are largely redundant [24,25,26]. However, targeted disruption of Cnot7 and Cnot8 leads to different phenotypes in mice, indicating that these paralogues are not fully redundant in all cell types. Inactivation of Cnot8 is embryonically lethal [26]. By contrast, Cnot7 knockout mice are viable and appear normal, but have several phenotypes, including defective spermatogenesis, resistance to diet-induced obesity, and increased bone mass due to enhanced osteoblast activity [27,28,29,30]. Cnot7 has also been implicated in the promotion of breast cancer metastasis, which is dependent on its nuclease activity [31].

Because the contributions of the Caf1 and Ccr4 subunits are not fully understood, we set out to develop Caf1/CNOT7 inhibitors. Such inhibitors are useful to dissect the catalytic and structural roles of the protein. To facilitate the discovery and development of Caf1/CNOT7 inhibitors, we previously developed a fluorescence-based assay that is suitable for the quantitative analysis of deadenylase activity [32]. This assay was used in combination with virtual screening for the discovery of substituted 5-(2-hydroxybenzoyl)-2-pyridone analogues as inhibitors of the human Caf1/CNOT7 ribonuclease [32,33]. In addition, the assay was employed for the development of a series of 1-hydroxy xanthine analogues, with the most potent inhibitor displaying micromolar activity towards Caf1/CNOT7 [34].

Because our previously identified inhibitors displayed limited potency, we aimed to identify new starting points for the development of more potent inhibitors. To identify new, structurally unrelated inhibitors, we set out to screen a library of 10,880 synthetic compounds that were selected based on structural diversity and drug-likeness using our previously described fluorescence-based assay. Using this approach, we discovered 15 drug-like inhibitors with IC_50_ values below 25 μM. A further six inhibitors were identified with IC_50_ values ranging from 50 to 230 μM. The compounds reported here may be useful as pharmacological tools to analyse the function of Caf1/CNOT7 as an alternative to genetic approaches, and are valuable starting points for the development of more potent inhibitors of Caf1/CNOT7 using medicinal chemistry.

## 2. Materials and Methods

### 2.1. Compound Screening

A representative subset of compounds (*n* = 10,880, purity > 95%) was selected from the Nottingham Managed Chemical Compound Collection. Compounds had the following profile: *MW*_r_ between 200 and 500, cLog *P* between 1 and 5, aromatic ring count between 2 and 3, <10 non-terminal rotatable bonds, between 2 and 6 H-bond acceptors, and <3 H-bond donors.

Compounds (10 mM in DMSO) were stored in a dry, inert environment at −20 °C in an automated sample management system (comPOUND, TTP Labtech Technology, Melbourn, UK). After dispensing aliquots in 96-well plates, compounds were diluted to 0.5 mM in 20% DMSO/water. Aliquots (4 mL; 25% DMSO/water) were transferred to black U-well 384-well plates (Greiner Bio-One, Kremsmünster, Austria) using a multichannel pipette. Subsequent screening was carried out as described before [32] with modifications for use with a Biomek 3000 liquid handler (Beckman Coulter, Brea, CA, USA). First, enzyme (8 μL containing 1.0 μM Caf1/CNOT7, 50 mM Tris–HCl pH 7.9, 125 mM NaCl, 5 mM MgCl_2_, 25% glycerol, 2.5 mM β-mercaptoethanol) was added. After mixing, the mixture was left at room temperature for 15 min before the addition of RNA substrate (8 μL; 0.25 μM Flc-RNA substrate in H_2_O). The composition of the final reaction mixture was: 0.4 μM Caf1/CNOT7, 0.1 μM Flc-RNA substrate, 20 mM Tris–HCl pH 7.9, 50 mM NaCl, 2 mM MgCl_2_, 10% glycerol, 1 mM β-mercaptoethanol, 5% DMSO, and 100 μM library compound. After incubation at 30 °C for 60 min, 20 μL probe mix (5 μM DNA probe, 1% SDS, 20 mM Tris–HCl pH 8.0, 0.5 mM EDTA) was added. Fluorescence was measured using a BioTek Synergy HT plate reader using the following filters: 485 ± 20 nm (excitation) and 528 ± 20 nm (emission). Hits were identified following data analysis in Microsoft Excel. Screening data was visualised using GraphPad Prism (version 10.4.0).

### 2.2. Determination of IC_50_ Values

Compounds were diluted in 25% DMSO/water typically assayed across eight data points ranging from 1|×|10^−6^ M to 1|×|10^−3^ M (final concentrations). Reaction conditions and fluorescence measurements were as described above and before [32]. Non-linear regression was carried out using GraphPad Prism 5.0. IC_50_ values were typically determined using three independent experiments, each containing three replicates.

### 2.3. Molecular Docking

Analysis of the active site of Caf1/CNOT7 was carried out using the DoGSiteScorer algorithm [35]. A structural model of human Caf1/CNOT7 was prepared for docking as described before [33]. Briefly, after removal of ions and water molecules, the human Caf1/CNOT7 protein was prepared for docking using the X-ray structure of Caf1/CNOT7 in complex with CNOT1 and Ccr4/CNOT6L (PDB 7VOI, chain B) by transposition of the Mg^2+^ ions in the active site of the *Schizosaccharomyces pombe* orthologue Pop2 (PDB 2P51) [16,36]. The addition of H atoms and conversion to pdbqt files was carried out using UCSF Chimera [37]. Ligands were prepared for docking using the SwissParam web portal (www.swissparam.ch) using the Merck Molecular ForceField [38,39]. Ligands were docked in the active site of CNOT7 using Autodock Vina [40,41] on the SwissDock portal (www.swissdock.ch; exhaustiveness 20) [42,43], using a grid of 25’25’25 Å^3^ centred around the active site Mg^2+^ ion coordinated by Asp-40, Glu-42, and Asp-230 (coordinates 136, 10, 270). Using the default scoring function, the top-scoring ligand poses were analysed using the Poseview algorithm accessed through the Proteins Plus portal to identify key interacting residues [44,45,46]. The receptor-ligand poses were visualised using PyMol [47].

## 3. Results and Discussion

**Figure 1:**
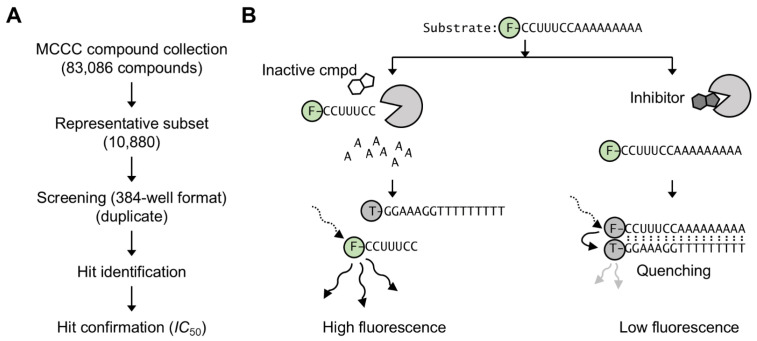
Compound screening strategy to identify inhibitors of the Caf1/CNOT7 poly(A)-selective nuclease. (**A**) Overview of screening strategy. A subset (10,880 compounds) of the Nottingham Managed Chemical Compound Collection was screened in duplicate using a 384-well plate format. The activity of hit compounds was confirmed by determining their IC_50_ values. (**B**) Fluorescence-based assay to identify inhibitors of Caf1/CNOT7 [32]. After incubation with inactive compounds (left), the fluorescein-labelled RNA substrate is degraded. After addition of the TAMRA-labelled DNA probe no annealing occurs, resulting in high fluorescence. In the presence of an inhibitor compound (right) the substrate is not degraded. After addition of the TAMRA-labelled DNA probe annealing takes place, resulting in low fluorescence due to quenching.

To discover new inhibitors of the Caf1/CNOT7 nuclease subunit of the Ccr4–Not complex, we screened a sub-set of the Nottingham Managed Chemical Compound Collection, which contains over 83,000 synthetic compounds that are commercially available and were selected based on chemical diversity and drug-like properties. A representative sub-set of 10,880 compounds was selected and screened in duplicate in a 384-well plate format (Figure 1A). The library compounds were screened using a fluorescence-based activity assay that was developed previously [32]. Briefly, purified Caf1/CNOT7 enzyme was pre-incubated with library compounds. This was followed by the addition of a 5′ fluorescein-labelled RNA oligonucleotide containing a stretch of nine adenosine residues at the 3′ end that served as substrate for Caf1/CNOT7. After incubation, the reaction was terminated and a 3′ TAMRA-labelled DNA probe complementary to the substrate was added. If the fluorescein-labelled substrate was degraded then the probe did not anneal, resulting in high substrate fluorescence. However, in case Caf1/CNOT7 was inhibited, the substrate remained intact, resulting in annealing of the DNA probe and quenching of fluorescence due to the close vicinity of the fluorescein and TAMRA fluorophores (Figure 1B).

Library compounds were screened at a fixed concentration (100 μM). Although batch-effects were noted (plates 1–28, 29–36, 37–52, and 53–68), high reproducibility was observed between hits in duplicate plates (Figure 2). The quality of the assay was also monitored during screening by calculating the Z’ factor for each plate, which varied between 0.8 and 0.5 (excellent) in the majority of cases (63/68). Five plates showed a Z’ factor of 0.4 (moderate). A relatively high number of compounds reduced activity of Caf1/CNOT7 to three standard deviations below the mean of the screening plate (107 compounds, 0.9% of total). Therefore, a more stringent threshold was used to select compounds for further analysis, and compounds were identified as hits if enzyme activity was inhibited to within 3 standard deviations of the negative control present in the same plate (Figure 2). Using this cut-off, 21 hits were identified (0.2% hit rate).

**Figure 3:**
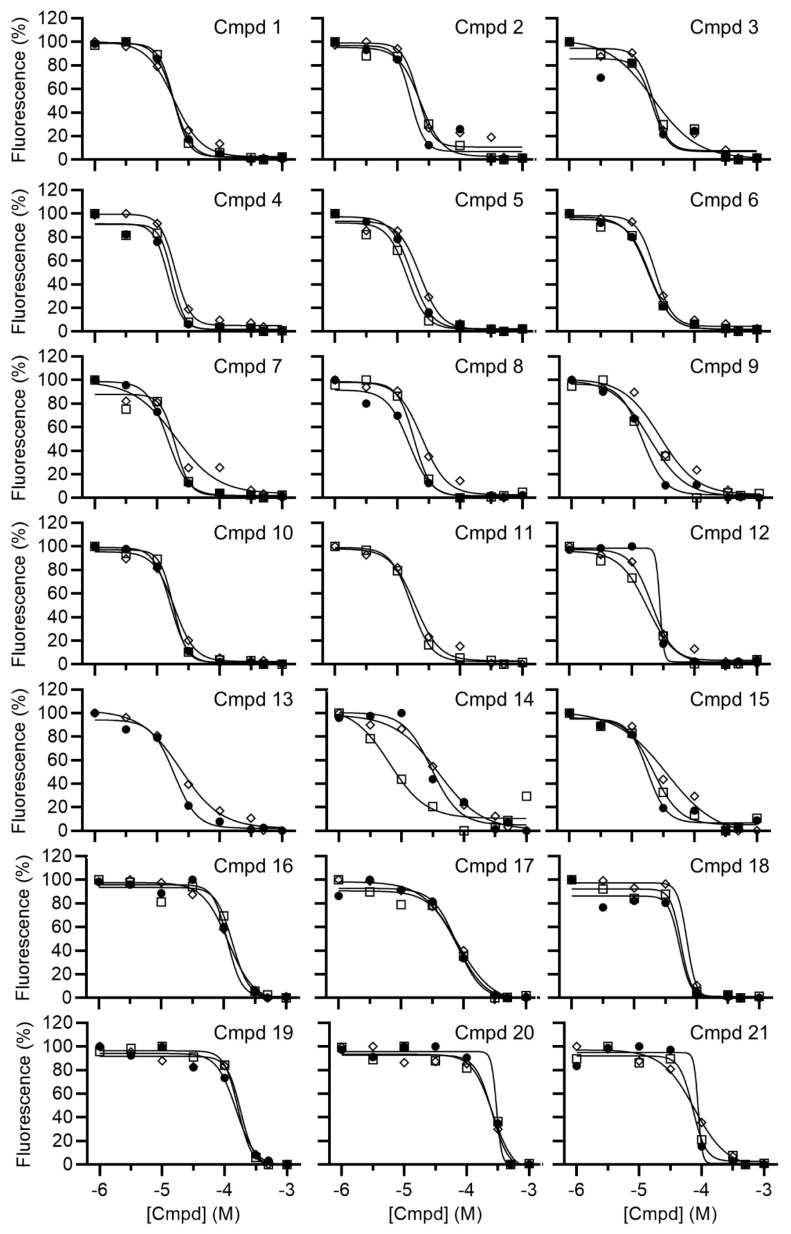
Determination of the IC_50_ values of small-molecule inhibitors of Caf1/CNOT7. Compounds were preincubated with Caf1/CNOT7 for 15 min at room temperature, followed by the addition of RNA substrate. After incubation (60 min at 30 °C), reactions were stopped by the addition of SDS and a 5-fold molar excess of DNA probe. The mean values for each independent experiment were plotted (Open square, experiment 1; closed circle, experiment 2; diamond, experiment 3).

**Figure 4:**
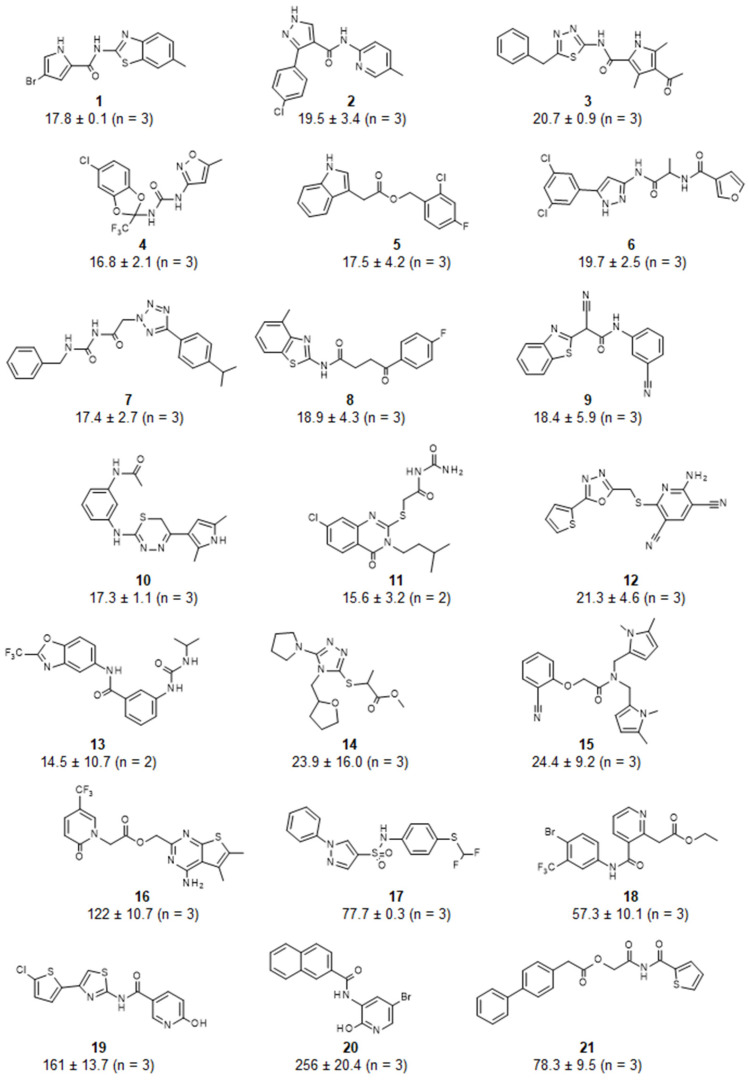
Structure and potency of inhibitors of the Caf1/CNOT7 poly(A)-selective ribonuclease. IC_50_ values were determined using a fluorescence-based assay described previously [32]. Reported are the mean ± standard deviation.

Following hit identification, the activity of the library compounds was confirmed by determining their potency. After establishing that the library compounds did not interfere with the fluorescence-based detection of the substrate, the IC_50_ values of the hits were determined using a dilution series of the compounds (Figure 3). Fifteen compounds were confirmed to have IC_50_ values <25 μM (Figure 4). A further three compounds displayed IC_50_ values below 100 μM, and three compounds had IC_50_ values between 122 and 256 μM. The predicted ADME profile [48] of all confirmed inhibitors indicated moderate/good solubility, cLogP < 4, compliance to Lipinski’s rule-of-five, high predicted gastrointestinal uptake (with exception of compounds 12 and 19), high predicted bioavailability, and the absence of pan-assay interference compounds (PAINSs) (Supplementary Data S1).

**Figure 5:**
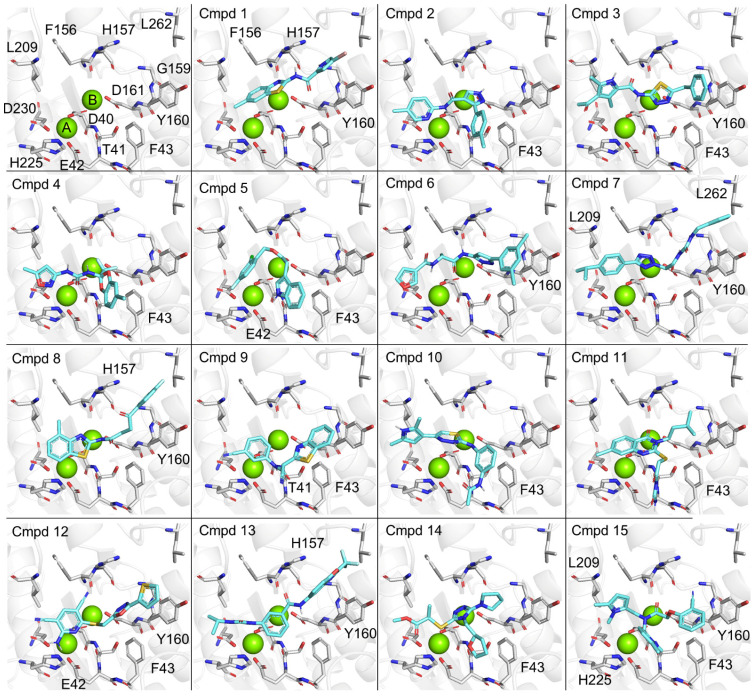
Possible binding modes of inhibitors in the active site of Caf1/CNOT7. Top left, active site of Caf1/CNOT7. Indicated are two Mg(II) ions (green) that are coordinated by residues Asp-40, Glu-42, Asp-161, and Asp-230. Possible binding modes of compounds 1–15 (cyan) in the active site of Caf1/CNOT7 are shown. Docking was carried out using Autodock Vina accessed via the SwissDock server [40,41,42,43]. Residues making key interactions as identified using the PoseView algorithm are indicated [45,46].

To obtain information about the possible binding modes of the identified inhibitors, we carried out molecular docking of the 15 compounds with IC_50_ values <25 μM. We used a docking approach that was described before, which was validated using docking of anative substrate of theenzyme (oligoA) that closely matched the original pose [33]. The active site of Caf1/CNOT7 is composed of a large cavity with a surface area of 742.6 Å^3^. The centre of the cavity contains two Mg^2+^ ions that are required for catalysis and coordinated by residues Asp-40, Glu-42, Asp-161, and Asp-230 (Figure 5, top left). Analysis of probable binding poses (Figure 5) suggested that Phe-43, Tyr-160, and the Mg(II) ions are of particular importance for ligand binding. Phe-43 forms probable π–π interactions with 2, 4, 9, 12, and 15, hydrophobic contacts with compounds 1–3, 5, 10, 9, 12, and 15, and backbone interactions as a H-bond acceptor mediated by the C=O moiety (10) and a H-bond donor via the N-H moiety (11). Tyr-160 forms probable π–π interactions with compounds 1, 3, 6–8, 12, 13 and 15, and additional hydrophobic contacts with compounds 3, 6–8, and 13. The Mg(II) ions were involved in numerous probable salt–metal (2–4, 7–9, 11, 13–14), cation–π (4–7, 11–12, 14–15) and metal–sulfur (8 and 12) interactions. Furthermore, probable H-bonds were identified between the backbone C=O of Thr-41 and 9, and H-bonding mediated by the side chains of Glu-42 and 5 and 12, and His-157 and 1. Further probable contacts involved hydrophobic interactions between Phe-156 and 1, His-157 and 8 and 13, Leu-209 and 7 and 15, His-225 and 15, and Leu-262 and 7. The possible interactions of the inhibitors reported here with residues Phe-43, Tyr-160, and a Mg(II) ion are interesting, because the probable binding pose of our recently reported inhibitors also interact with these residues of Caf1/CNOT7 [33].

## 4. Conclusions

The work reported here indicates that the fluorescence-based nuclease assay reported previously [32] is robust (Z’ > 0.5 in 63/68 384-well plates) and suitable for screening large (>10,000) numbers of compounds using 384-well plates and automated robotic liquid handling systems. The compound screening resulted in the discovery of 21 new inhibitors of Caf1/CNOT7, including 15 drug-like compounds with IC_50_ values below 25 μM. The compounds reported here are commercially available and can be used in biochemical experiments as inhibitors of Caf1/CNOT7 and as starting points for the development of more potent and cell-permeable inhibitors that inhibit Caf1/CNOT7. Such inhibitors will be useful tools to complement genetic approaches for the further investigation of the role of Caf1/CNOT7 in complex cellular processes and diseases such as cancer metastasis and bone formation.

## Figures and Tables

**Figure 2:**
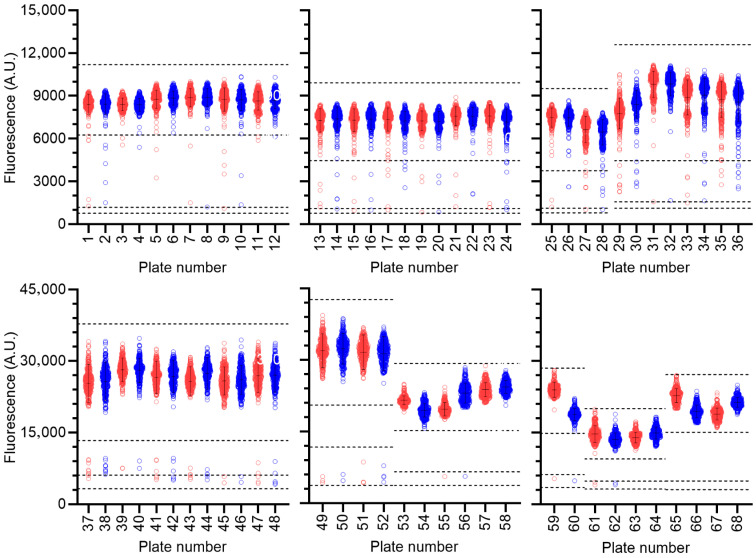
Compound screening. Compounds (*n* = 10,880) were screened in duplicate using 320 library compounds per plate. Duplicate plates are indicated in red (odd numbers) and blue (even numbers), respectively. Error bars represent the mean and standard deviation of wells containing library compounds for each plate. Dotted lines represent largest 3’ standard deviations of the wells containing library compounds and negative control ± 3’ standard deviations.

## Data Availability

The original contributions presented in this study are included in the article/Supplementary Material. Further inquiries can be directed to the corresponding author(s).

## Supplementary Materials

The following supporting information can be downloaded at: https://www.mdpi.com/article/10.3390/biom15111563/s1, Supplementary Data S1, Predicted ADME profile.

## References

[1] Collart M.A., Panasenko O.O. (2017). The Ccr4-Not Complex: Architecture and Structural Insights. Subcell Biochem..

[2] Pavanello L., Hall M., Winkler G.S. (2023). Regulation of eukaryotic mRNA deadenylation and degradation by the Ccr4-Not complex. Front. Cell Dev. Biol..

[3] Raisch T., Valkov E. (2022). Regulation of the multisubunit CCR4-NOT deadenylase in the initiation of mRNA degradation. Curr. Opin. Struct. Biol..

[4] Krempl C., Lazzaretti D., Sprangers R. (2023). A structural biology view on the enzymes involved in eukaryotic mRNA turnover. Biol. Chem..

[5] Muller M.B.D., Becker T., Denk T., Hashimoto S., Inada T., Beckmann R. (2025). The ribosome as a platform to coordinate mRNA decay. Nucleic Acids Res..

[6] Absmeier E., Chandrasekaran V., O’Reilly F.J., Stowell J.A.W., Rappsilber J., Passmore L.A. (2023). Specific recognition and ubiquitination of translating ribosomes by mammalian CCR4-NOT. Nat. Struct. Mol. Biol..

[7] Buschauer R., Matsuo Y., Sugiyama T., Chen Y.H., Alhusaini N., Sweet T., Ikeuchi K., Cheng J., Matsuki Y., Nobuta R. (2020). The Ccr4-Not complex monitors the translating ribosome for codon optimality. Science.

[8] Zhu X., Cruz V.E., Zhang H., Erzberger J.P., Mendell J.T. (2024). Specific tRNAs promote mRNA decay by recruiting the CCR4-NOT complex to translating ribosomes. Science.

[9] Goldstrohm A.C., Wickens M. (2008). Multifunctional deadenylase complexes diversify mRNA control. Nat. Rev. Mol. Cell Biol..

[10] Parker R., Sheth U. (2007). P bodies and the control of mRNA translation and degradation. Mol. Cell.

[11] Wahle E., Winkler G.S. (2013). RNA decay machines: Deadenylation by the Ccr4-Not and Pan2-Pan3 complexes. Biochim. Biophys. Acta.

[12] Bianchin C., Mauxion F., Sentis S., Seraphin B., Corbo L. (2005). Conservation of the deadenylase activity of proteins of the Caf1 family in human. RNA.

[13] Horiuchi M., Takeuchi K., Noda N., Muroya N., Suzuki T., Nakamura T., Kawamura-Tsuzuku J., Takahasi K., Yamamoto T., Inagaki F. (2009). Structural basis for the antiproliferative activity of the Tob-hCaf1 complex. J. Biol. Chem..

[14] Basquin J., Roudko V.V., Rode M., Basquin C., Seraphin B., Conti E. (2012). Architecture of the nuclease module of the yeast Ccr4-not complex: The Not1-Caf1-Ccr4 interaction. Mol. Cell.

[15] Dupressoir A., Morel A.P., Barbot W., Loireau M.P., Corbo L., Heidmann T. (2001). Identification of four families of yCCR4- and Mg^2+^-dependent endonuclease-related proteins in higher eukaryotes, and characterization of orthologs of yCCR4 with a conserved leucine-rich repeat essential for hCAF1/hPOP2 binding. BMC Genom..

[16] Zhang Q., Pavanello L., Potapov A., Bartlam M., Winkler G.S. (2022). Structure of the human Ccr4-Not nuclease module using X-ray crystallography and electron paramagnetic resonance spectroscopy distance measurements. Protein Sci..

[17] Wang H., Morita M., Yang X., Suzuki T., Yang W., Wang J., Ito K., Wang Q., Zhao C., Bartlam M. (2010). Crystal structure of the human CNOT6L nuclease domain reveals strict poly(A) substrate specificity. EMBO J..

[18] Chen Y., Khazina E., Izaurralde E., Weichenrieder O. (2021). Crystal structure and functional properties of the human CCR4-CAF1 deadenylase complex. Nucleic Acids Res..

[19] Maryati M., Airhihen B., Winkler G.S. (2015). The enzyme activities of Caf1 and Ccr4 are both required for deadenylation by the human Ccr4-Not nuclease module. Biochem. J..

[20] Pekovic F., Rammelt C., Kubikova J., Metz J., Jeske M., Wahle E. (2023). RNA binding proteins Smaug and Cup induce CCR4-NOT-dependent deadenylation of the nanos mRNA in a reconstituted system. Nucleic Acids Res..

[21] Webster M.W., Chen Y.H., Stowell J.A.W., Alhusaini N., Sweet T., Graveley B.R., Coller J., Passmore L.A. (2018). mRNA Deadenylation Is Coupled to Translation Rates by the Differential Activities of Ccr4-Not Nucleases. Mol. Cell.

[22] Yi H., Park J., Ha M., Lim J., Chang H., Kim V.N. (2018). PABP Cooperates with the CCR4-NOT Complex to Promote mRNA Deadenylation and Block Precocious Decay. Mol. Cell.

[23] Winkler G.S., Balacco D.L. (2013). Heterogeneity and complexity within the nuclease module of the Ccr4-Not complex. Front. Genet..

[24] Aslam A., Mittal S., Koch F., Andrau J.C., Winkler G.S. (2009). The Ccr4-Not Deadenylase Subunits CNOT7 and CNOT8 Have Overlapping Roles and Modulate Cell Proliferation. Mol. Biol. Cell.

[25] Mittal S., Aslam A., Doidge R., Medica R., Winkler G.S. (2011). The Ccr4a (CNOT6) and Ccr4b (CNOT6L) deadenylase subunits of the human Ccr4-Not complex contribute to the prevention of cell death and senescence. Mol. Biol. Cell.

[26] Mostafa D., Takahashi A., Yanagiya A., Yamaguchi T., Abe T., Kureha T., Kuba K., Kanegae Y., Furuta Y., Yamamoto T. (2020). Essential functions of the CNOT7/8 catalytic subunits of the CCR4-NOT complex in mRNA regulation and cell viability. RNA Biol..

[27] Berthet C., Morera A.M., Asensio M.J., Chauvin M.A., Morel A.P., Dijoud F., Magaud J.P., Durand P., Rouault J.P. (2004). CCR4-associated factor CAF1 is an essential factor for spermatogenesis. Mol. Cell. Biol..

[28] Nakamura T., Yao R., Ogawa T., Suzuki T., Ito C., Tsunekawa N., Inoue K., Ajima R., Miyasaka T., Yoshida Y. (2004). Oligo-astheno-teratozoospermia in mice lacking Cnot7, a regulator of retinoid X receptor beta. Nat. Genet..

[29] Takahashi A., Adachi S., Morita M., Tokumasu M., Natsume T., Suzuki T., Yamamoto T. (2015). Post-transcriptional Stabilization of Ucp1 mRNA Protects Mice from Diet-Induced Obesity. Cell Rep..

[30] Washio-Oikawa K., Nakamura T., Usui M., Yoneda M., Ezura Y., Ishikawa I., Nakashima K., Noda T., Yamamoto T., Noda M. (2007). Cnot7-null mice exhibit high bone mass phenotype and modulation of BMP actions. J. Bone Min. Res.

[31] Faraji F., Hu Y., Yang H.H., Lee M.P., Winkler G.S., Hafner M., Hunter K.W. (2016). Post-transcriptional Control of Tumor Cell Autonomous Metastatic Potential by CCR4-NOT Deadenylase CNOT7. PLoS Genet..

[32] Maryati M., Kaur I., Gopal J., Olotu-Umoren L., Oveh B., Hashmi L., Fischer P.M., Winkler G.S. (2014). A fluorescence-based assay suitable for quantitative analysis of deadenylase enzyme activity. Nucleic Acids Res..

[33] Kaur I., Jadhav G.P., Fischer P.M., Winkler G.S. (2024). Discovery of Substituted 5-(2-Hydroxybenzoyl)-2-Pyridone Analogues as Inhibitors of the Human Caf1/CNOT7 Ribonuclease. Molecules.

[34] Jadhav G.P., Kaur I., Maryati M., Airhihen B., Fischer P.M., Winkler G.S. (2015). Discovery, synthesis and biochemical profiling of purine-2,6-dione derivatives as inhibitors of the human poly(A)-selective ribonuclease Caf1. Bioorganic Med. Chem. Lett..

[35] Volkamer A., Kuhn D., Rippmann F., Rarey M. (2012). DoGSiteScorer: A web server for automatic binding site prediction, analysis and druggability assessment. Bioinformatics.

[36] Jonstrup A.T., Andersen K.R., Van L.B., Brodersen D.E. (2007). The 1.4-A crystal structure of the S. pombe Pop2p deadenylase subunit unveils the configuration of an active enzyme. Nucleic Acids Res..

[37] Pettersen E.F., Goddard T.D., Huang C.C., Couch G.S., Greenblatt D.M., Meng E.C., Ferrin T.E. (2004). UCSF Chimera--a visualization system for exploratory research and analysis. J. Comput. Chem..

[38] Bugnon M., Goullieux M., Rohrig U.F., Perez M.A.S., Daina A., Michielin O., Zoete V. (2023). SwissParam 2023: A Modern Web-Based Tool for Efficient Small Molecule Parametrization. J. Chem. Inf. Model..

[39] Zoete V., Cuendet M.A., Grosdidier A., Michielin O. (2011). SwissParam: A fast force field generation tool for small organic molecules. J. Comput. Chem..

[40] Eberhardt J., Santos-Martins D., Tillack A.F., Forli S. (2021). AutoDock Vina 1.2.0: New Docking Methods, Expanded Force Field, and Python Bindings. J. Chem. Inf. Model..

[41] Trott O., Olson A.J. (2010). AutoDock Vina: Improving the speed and accuracy of docking with a new scoring function, efficient optimization, and multithreading. J. Comput. Chem..

[42] Bugnon M., Rohrig U.F., Goullieux M., Perez M.A.S., Daina A., Michielin O., Zoete V. (2024). SwissDock 2024: Major enhancements for small-molecule docking with Attracting Cavities and AutoDock Vina. Nucleic Acids Res..

[43] Grosdidier A., Zoete V., Michielin O. (2011). SwissDock, a protein-small molecule docking web service based on EADock DSS. Nucleic Acids Res..

[44] Schoning-Stierand K., Diedrich K., Fahrrolfes R., Flachsenberg F., Meyder A., Nittinger E., Steinegger R., Rarey M. (2020). ProteinsPlus: Interactive analysis of protein-ligand binding interfaces. Nucleic Acids Res..

[45] Stierand K., Rarey M. (2010). Drawing the PDB: Protein-Ligand Complexes in Two Dimensions. ACS Med. Chem. Lett..

[46] Stierand K., Maass P.C., Rarey M. (2006). Molecular complexes at a glance: Automated generation of two-dimensional complex diagrams. Bioinformatics.

[47] Schrödinger L.L.C. (2025). The PyMol Molecular Graphics System.

[48] Daina A., Michielin O., Zoete V. (2017). SwissADME: A free web tool to evaluate pharmacokinetics, drug-likeness and medicinal chemistry friendliness of small molecules. Sci. Rep..

